# Insecticide-treated eave ribbons for malaria vector control in low-income communities

**DOI:** 10.1186/s12936-021-03945-2

**Published:** 2021-10-23

**Authors:** Emmanuel W. Kaindoa, Arnold S. Mmbando, Ruth Shirima, Emmanuel E. Hape, Fredros O. Okumu

**Affiliations:** 1 Environmental Health and Ecological Science Department, Ifakara Health Institute, P. O. Box 53, Ifakara, Tanzania; 2 Department of Biosciences, Durham University, DH13LE Durham, UK; 3 School of Public Health, Faculty of Health Sciences, University of the Witwatersrand, Johannesburg, South Africa; 4 Institute of Biodiversity, Animal Health and Comparative Medicine, University of Glasgow, G12 8QQ Glasgow, UK; 5 School of Life Science and Bioengineering, The Nelson Mandela African Institution of Science and Technology, P. O. Box 447, Arusha, Tanzania

**Keywords:** Eave ribbons, Spatial repellents, Malaria, Indoor residual spraying

## Abstract

Supplementary tools are required to address the limitations of insecticide-treated nets (ITNs) and indoor residual spraying (IRS), which are currently the core vector control methods against malaria in Africa. The eave ribbons technology exploits the natural house-entry behaviours of major malaria vectors to deliver mosquitocidal or repellent actives around eave spaces through which the *Anopheles* mosquitoes usually enter human dwellings. They confer protection by preventing biting indoors and in the peri-domestic outdoor spaces, and also killing a significant proportion of the mosquitoes. Current versions of eave ribbons are made of low-cost hessian fabric infused with candidate insecticides and can be easily fitted onto multiple house types without any additional modifications. This article reviews the evidence for efficacy of the technology, and discusses its potential as affordable and versatile supplementary approach for targeted and efficient control of mosquito-borne diseases, particularly malaria. Given their simplicity and demonstrated potential in previous studies, future research should investigate ways to optimize scalability and effectiveness of the ribbons. It is also important to assess whether the ribbons may constitute a less-cumbersome, but more affordable substitute for other interventions, such as IRS, by judiciously using lower quantities of selected insecticides targeted around eave spaces to deliver equivalent or greater suppression of malaria transmission.

## Background

Malaria deaths declined by 60% between the year 2000 and 2019 [1], and by more than 50% in some high-burden countries, such as Tanzania [2]. These gains resulted primarily from scale up of three main interventions, namely insecticide-treated nets (ITNs), indoor residual-sprays (IRSs) and improved case management [2–5]. In addition, malaria endemic countries may have benefited also from improved access to health care, as well as the overall economic growth and urbanization [6]. A recent analysis of progress towards the targets set in the World Health Organization (WHO) Global Technical Strategy for Malaria (GTS 2016–2030) [7] indicated that the 2020 goals of reducing incidence and mortality were already missed by 37% and 22%, respectively [1].

Despite the observed successes, the protective efficacies of ITNs and IRS are threatened by multiple factors, the most commonly discussed being, widespread pyrethroid resistance [8–10], increased outdoor-biting [11], early biting especially indoors [12] and some exposure-prone human activities and behaviours [13]. ITNs and IRS will be inadequate for malaria elimination, and additional approaches are urgently necessary to tackle the key challenges [14].

As malaria control progresses, the populations most affected increasingly consist of households in rural and peri-urban communities, particularly those living in poorly-constructed houses with gaps on roofs, eaves, walls, windows and doors [15–18]. One meta-analysis showed that compared to traditional houses, residents of modern homes may have 45–65% lower odds of getting clinical malaria [19]. Whereas low-income households cannot always afford the essential home improvements [20], experimental evidence shows that house designs significantly affect indoor densities of *Anopheles* mosquitoes and overall malaria transmission in Africa [21–25]. Past studies have assessed how mosquitoes enter human houses [16, 17, 23, 24], showing that improved understanding of host-seeking behaviours and the associated household factors are important in designing vector control methods.

Malaria elimination programmes must seek long-term environmental and health-system resilience to sustain the gains accrued from current commodities, namely drugs, diagnostics, mosquito nets and insecticides. In the meantime, countries may adopt additional methods to effectively complement ITNs and IRS. Examples may include larviciding [26] and house screening [18]. There are also a number of promising tools under development or evaluation, including attractive targeted sugar baits (ATSBs), spatial repellents (SR), topical repellents [27], endectocides [28], odour-baited traps (OBTs), and use of genetically modified mosquitoes [29]. Since host-seeking *Anopheles* mosquitoes typically spend significant lengths of time close to the eaves before eventually entering houses [30], there are also a number of eave-based technologies, aimed at addressing current control gaps by targeting mosquitoes entering homes via the eaves space. Key examples include lethal house lures incorporating eave tubes [31, 32], insecticide-treated curtains [33, 34] or ceilings [35], as well as insecticide-treated eave baffles [36] and eave ribbons [37].

The eave ribbons approach, recently developed by Ifakara Health Institute, exploits the same mosquito behaviours as other eave-based technologies, notably eave curtains and eave baffles [33, 36, 38], but induces both spatial repellence and mosquito mortality to protect users indoors and outdoors [37]. It carries the additional advantage of being simple and highly scalable, and can be fitted even in the poorest dwellings as well as itinerant homes [39]. As such, it has been proposed as one tool that could enable judicious application of available or new insecticide classes in ways that maximize the control of vector-borne pathogens even in the lowest-income communities.

This article reviews the design features, evidence for efficacy, plausible development pathways and the future potential of eave ribbons as a method to achieve targeted and efficient control of mosquito-borne diseases, notably malaria. The paper focuses primarily on insecticide-treated ribbons used on the outer surfaces of eaves, and is not intended to cover all eave-based technologies.

### Design features

**Fig. 1:**
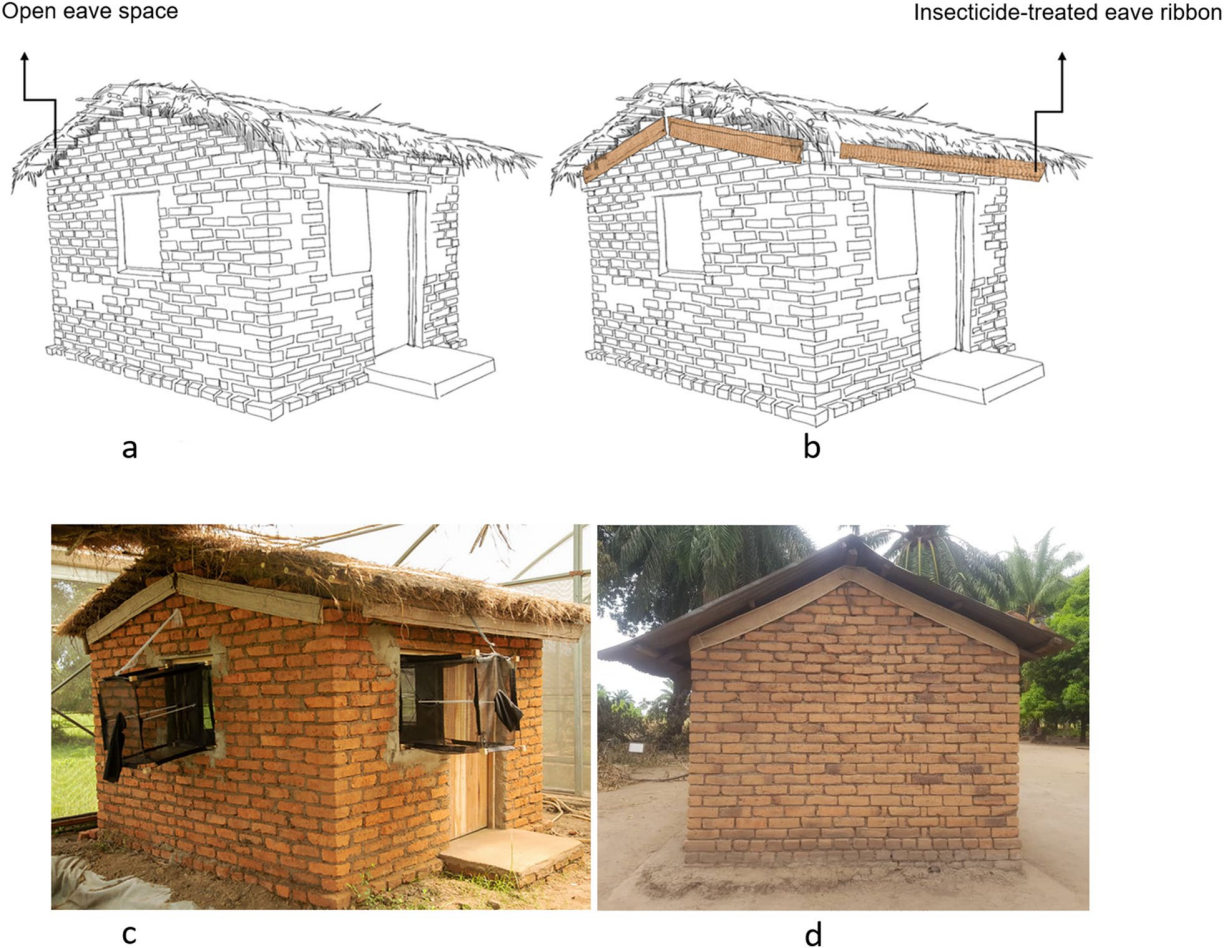
Illustration of houses with and without insecticide-treated eave ribbons (**a**, **b**). Also shown are ribbons affixed to an experimental hut during scientific evaluation (**c**), and to a local housing structure used by migratory farmers in rural Tanzania (**d**). The eave ribbons may constitute practical and affordable protection options suitable for all house types irrespective of design, and are used as a delivery method for proven insecticidal treatments

Current eave ribbons are made of a 15 cm-wide double or triple-layered hessian fabric, weighing approximately 500 g m^−2^ and has varying lengths depending on target house size. The initial studies done in the semi field, the eave ribbons measured 0.15 m wide by 2.5 m long, while tests done in the field used ribbons measuring 0.15 m wide by 25 m long. The eave ribbons present a novel deployment method for a range of products, including vapour-phase and contact insecticides. The hessian material was sourced locally in East Africa, where the material is used for manufacturing a wide range of other products, such as sacks, rope and decorations. The ribbons are fitted onto houses using nails, adhesives or other fasteners, without completely closing eave spaces (Fig. 1). More detailed descriptions of the ribbons can be found in Mmbando et al. [37], Mwanga et al. [40] and Swai et al. [39]. The ribbons can be fixed into poorly-constructed houses with gaps on eaves, doors or walls (Fig. 1). No house modifications or electricity are necessary to affix the ribbons [39].

### Development and experimental evaluation of eave ribbons

The eave ribbons technology evolved from early evaluations of spatial repellents conducted at Ifakara Health Institute under semi-field systems [41] and in rural Tanzanian villages using either experimental huts or local homes [41–43]. By design, it is a variant of previous eave-based technologies notably the insecticide treated eave curtains [33, 38], and insecticide treated eave-baffles [36], but is designed for outdoor placement and aimed at conferring protection both indoors and outdoors.

The early studies, leading to the development of eave-ribbons technology, mainly investigated new methods for delivering transfluthrin and other candidate spatial repellents. Following initial studies by Ogoma et al. [41], Mmbando et al. demonstrated the use of hessian ribbons along eave spaces in experimental huts; where the ribbons were used alone [37, 44] or in combination with odour-baited mosquito traps in push-pull systems [44]. The ribbons were affixed onto volunteer occupied huts, and mosquitoes trapped indoors from 10 pm to 6 am and in the peri-domestic space from 6 pm to 10 pm using exposure-free methods. The studies demonstrated significant protection both indoors and outdoors, against the major malaria vectors, *Anopheles arabiensis* and *Anopheles funestus*, despite being resistant to pyrethroids.

Follow-up studies have since also demonstrated 77% protection against *An. arabiensis*, 60% against *An. funestus* and 98% against *Culex* spp. in the migratory farming communities in Tanzania, where the ribbons were fitted around the semi-open makeshift structures used by the itinerant famers in distant river valleys [39]. Many of these farmers dwell in semi-open poorly constructed structures, yet the ribbons could be readily fitted without any prior house modification. There have also been studies evaluating other transfluthrin-treated hessian emanators [42], chairs [45], artistic decorations [46] and sandals [47]. A combined analysis of these early studies shows that the transfluthrin-treated hessian products could: (i) retain protective efficacy for 6 months or more [42], (ii) be readily acceptable to local communities [39, 46], (iii) be made locally without specialized skills needed, (iv) offer protection against indoor and outdoor mosquito bites [37, 43], (v) have mosquitocidal effects [toxicity] in addition to repellency [48], and (vi) protect against multiple mosquito species including pyrethroid-resistant *Anopheles* [45]. The studies also showed that when used together with traps in push-pull systems, the protective benefit came primarily from the treated eave ribbons themselves [44].

Most of these studies examined protection at the level of individual households. However, one semi-field experiment also demonstrated communal protection for both the users and non-users of the ribbons, especially once the proportion of user households exceeded 60% [40]. More recently, small scale field studies in rural Tanzania have demonstrated entomological benefits in villages with eave ribbons compared to control villages (Mmbando et al., pers. commun.). Though there has not been large-scale epidemiological trials, experimental studies and mathematical models have shown efficacy of transfluthrin-treated eave ribbons to reduce biting both indoors and outdoors, and potential to disrupt overall malaria transmission [49]. Table 1 summarises the studies on eave ribbons.

**Table 1:** Examples of studies on eave ribbons treated with transfluthrin, demonstrating effects on mosquito densities and biting rates inside and outside human dwellings

SN	Study	Methods details	Mosquitoes	% Mortality	% Biting reduction	Conclusions	References
1	Semi field evaluation of the effectiveness of transfluthrin- treated hessian fabric against malaria vector vectors	Hessian strip [4 m × 30 cm] impregnated with 10 ml technical grade transfluthrin prepared in soapy water; the strips suspended overnight indoors to dry Tests were done in large semi-field cage	Laboratory-reared *Anopheles arabiensis*	Not assessed	99%	Hessian materials are affordable, locally produced and are very efficient in delivering transfluthrin vapour in to space to offer protection against mosquito bites	Ogoma et al. [41]
2	Field study to quantify the protective efficacy of transfluthrin treated hessian strips against outdoor-biting *Anopheles gambiae* s.l. and *Culex* mosquitoes	Hessian strip [4 m × 30 cm] impregnated with 10 ml technical grade transfluthrin prepared in soapy water; the strips suspended overnight indoors to dry Tests were done in the field	Wild *Anopheles gambiae* s.l. and *Culex* spp.	Not assessed	99% [*Anopheles gambiae* s.l.]	No diversion of mosquitoes to non-users Significant protection against outdoor biting mosquitoes	Govella et al. [43]
3	To measure the durability and long term efficacy of transfluthrin treated hessian strips	Hessian strip [4 m × 30 cm] impregnated with 10 ml technical grade transfluthrin prepared in soapy water; the strips suspended overnight indoors to dry Tests were done in the field	Wild *Anopheles gambiae* s.l. and *Culex* spp.	Not assessed	> 90%	Transfluthrin-treated hessian emanators provide safe and long-term protection against outdoor biting Also protected nearby non-users Concentration of transfluthrin in an enclosed room was 3 times less than the recommended threshold, hence ensuring maximum safety to users	Ogoma et al. [42]
4	To assess the protective efficacy and acceptability of the different hessian material designs treated with transfluthrin used in in rural communities	Hessian strip [0.28 m^2^] impregnated with 5ml technical grade transfluthrin prepared in soapy water; the strips suspended overnight indoors to dry Tests were done in the field	Wild *Anopheles* mosquitoes and *Culex* spp	Not assessed	86–89% [*An. arabiensis*]	The treated hessian materials are widely accepted by community members Transfluthrin-treated hessian materials provide significant protection against outdoor-biting malaria vectors	Masalu et al. [46]
5	Assess the protective efficacy of transfluthrin treated eave ribbons against indoor and outdoor mosquito bites	The eave ribbons were made of triple-layered hessian fabric woven using sisal fibres The ribbons used here were either 15 cm wide and 2.5 m long (for fitting onto the front and back sides of the huts) or 15 cm wide and 1 m long (one pair for fitting on the right side and another pair for the left-side of the huts) Treatments were done following the procedures described by Ogoma et al.	Laboratory- reared *Anopheles arabiensis* Wild *Anopheles* mosquitoes and *Culex* spp.	99.5%	99%	Eave ribbons provide protection to not only indoor but also outdoors	Mmbando et al. [37]
6	To evaluate the efficacy of transfluthrin-treated eave ribbons in protecting rural migratory farmers in in rural Tanzania	Eave ribbons were treated following the procedures described by Mmbando et al.	Wild *Anopheles* mosquitoes and *Culex* spp.	Not assessed	77%	The eave ribbons are highly acceptable by community members Transfluthrin-treated eave ribbons could be used in addition to ITNs to protect migratory people at high risk of malaria transmission Eave ribbons also protected against both indoor and outdoor biting *Anopheles* and *Culex* mosquitoes	Swai et al. [39]
7	To assess the protective efficacy of treated eave ribbons provided to users and non-users	Eave ribbons were treated following the procedures described by Mmbando et al. [37]	*Laboratory- reared Anopheles arabiensis*	100%	83% indoors for users 62% outdoors for users 57% indoors for non-users 48% outdoors non-users	Eave ribbon provide protection even to non-users The 100% mortality observed at 24 h implies that there is a potential for eave ribbons to have a communal mass effect due to its killing effect, thereby crushing vector population as well as vectorial capacity	Mwanga et al. [40]
8	To assess the additional benefits of combining treated eave ribbons with mosquito traps	Eave ribbons were treated following the procedures described by Mmbando et al. [37]	Laboratory-reared *Anopheles arabiensis*	Not assessed	81.2% indoor 63% outdoor	Eave ribbons provide sufficient protection, thus no need of additional traps	Mmbando et al. [44]
9	To assess protective efficacy of hessian fabric mats and ribbons treated against mosquitoes	Eave ribbons were treated following the procedures described by Mmbando et al. [37]	Wild mosquito population	100%	77–81.2%	There was 100% mortality of *An. funestus* mosquitoes which are known to be resistant to pyrethroids. Hence eave ribbons have potential in managing insecticide resistance	Masalu et al. [45]
10	Predicting the impact of outdoor vector control interventions on malaria transmission intensity from semi-field studies	Statistical modelling of semi field eave ribbons data from Tanzania and Kenya	*Laboratory-reared Anopheles arabiensis*		41–96%	Transfluthrin treated eave ribbons provide both personal and community protection.	Denz et al. [49]
11	Transfluthrin Eave-Positioned Targeted Insecticide (EPTI) Reduces Human Landing Rate of Pyrethroid Resistant and Susceptible Malaria Vectors in a Semi Field Simulated Peri-domestic Space	Eave ribbons were treated following the procedures described by Mmbando et al. [37]	*Laboratory-reared Anopheles arabiensis*	80%	68%	Transfluthrin treated eave ribbons have potential tackle the challenges of insecticide resistance in malaria vectors	Tambwe et al. [56]
12	Evaluating putative repellent ‘push’ and attractive ‘pull’ components for manipulating the odour orientation of host-seeking malaria vectors in the peri-domestic space	Hessian strip [21 m × 0.05 cm] impregnated with 10 ml technical grade transfluthrin prepared in soapy water; the strips suspended overnight indoors to dry Tests were done in large semi-field cage	Laboratory-reared *Anopheles arabiensis*	Not assessed	94%	Transfluthrin treated hessian fabric strips around eave gaps provide significant protection from mosquito bites in the peri-domestic spaces.	Njoroge et al. [57]

The table focuses primarily on hessian-based eave ribbons and excludes other eave-based technologies

### Potential for insecticide resistance management

Insecticide resistance is attenuating protective power of insecticidal approaches, thus novel approaches are urgently needed to overcome this challenge [50–52]. The eave ribbon technology could be amenable to treatment with a wide variety of insecticide classes, and could be relied upon to enhance efforts for resistance management. For this, the actual treatments on eave ribbons may include chemicals already approved for IRS, including organophosphates, organochlorines or neonicotinoids [53]. Studies should be done to assess the potential impact of using eave ribbons treated with different chemical classes or their combinations for managing resistance, and the performance of such ribbons when used simultaneously with other vector control tools, notably ITNs.

Though transfluthrin, currently the only active used on eave ribbon studies, is also a pyrethroid, evidence suggests that it can remain effective against mosquitoes with Cytochrome P450-mediated metabolic resistance. A possible explanation is that the chemical structure of transfluthrin is functionally different from other pyrethroids, hence the P450 detoxifying enzymes are less effective [54]. One recent study, done in an area with widespread P450-mediated pyrethroid-resistance [55], showed 99.4–100% mortality in *An. arabiensis* and *An. funestus* exposed under transfluthrin-treated chairs [45]. Future research should investigate the functional efficacy of transfluthrin used alone or in combination with other pesticides in areas with confirmed resistance to other pyrethroids commonly used in public health.

### Need for expanded evaluation of eave ribbons technology for malaria vector control and associated safety requirements

The evidence outlined in Table 2 suggests that transfluthrin treated ribbons have potential as complementary tools for controlling disease-transmitting mosquitoes. However, additional studies are required to validate their performance in disease endemic communities. The studies should assess entomological and epidemiological benefits, address questions of delivery and retreatment, compare cost-effectiveness of the technology to other interventions, monitor perceptions of target users and assess the degree of acceptability for this technology.

**Table 2:** Some challenges associated with standard IRS practices, and potential of pre-treated eave ribbons to address these challenges

Attributes	Challenges associated with standard IRS practices	Potential of insecticide- eave ribbons to address the IRS-related challenges
Quantities of chemicals	Large quantities of chemicals may be needed to treat all indoor surfaces	Significantly reduced quantities of chemicals will be required to treat the ribbons [36]
Spraying operations	Requires removal of household belongings before spraying; this slows down operations and can limit acceptability	Will not require removal of household belongings, thus can be done rapidly and at scale
Implementation teams	Implementation requires large team of well-trained personnel	Implementation can be done by individuals and does not require spray teams
Scalability	Difficult to achieve large-scale coverage across regions or countries because of costs and logistical challenges	Wider coverage can be obtained once supply chain is established
Mosquitoes targeted	Targets mosquitoes spread out on indoor resting surfaces	Target mosquitoes at specific points of entry [37]
Target surface	IRS monitoring is sub-optimal given differences in substrates on people’s walls; varied indoor resting behaviours of mosquitoes [60], and post-spraying changes on sprayed surfaces [61]	Monitoring can be standardized since the treatment substrate is standardizable

The contents of this table are not meant to directly compare indoor residual spraying against and insecticide-treated eave ribbons. Instead, the objective is to identify specific IRS challenges that can potentially be addressed by using current or improved versions of insecticide-treated ribbons. Nonetheless, full determination of these attributes requires comparative field evaluation of insecticide-treated eave ribbons and standard IRS practices

Mathematical models, may also be used to map the target product profiles and guide further development. The key characteristics of the ribbons potentially allow deployment in multiple scenarios, including: (i) protecting low-income households in rural areas and urban slums, including those living in very poorly-constructed houses that cannot be readily screened or modified without being damaged; (ii) protecting people in the peri-domestic spaces, away from homes and indoors at times before bed net use; (iii) for protecting migratory populations such as itinerant farmers, forest workers and campers, pastoralists or fishing communities; (iv) protecting people in temporary shelters such as refugee camps, mining camps, or recreational sites; or (v) as a possible alternative for IRS especially if the ribbons can be safely pre-treated or treated on site and delivered at scale. All these areas need additional field data to validate actual potential and cost-effectiveness.

One study by Ogoma et al., which tested concentrations of transfluthrin emanating from the hessian treatments indoors, found that the residual air-borne quantities after 1 h exposure were undetectable using standard instruments for assessing air-quality [42]. Even after 24 h, the concentrations remained > 1000 times below the maximum acceptable concentration for long-term inhalation exposure of humans (500 µg m^−3^) defined by the regulatory authorities of the European Union (EU) [42]. Beyond the limited inhalation exposures, accidental physical contacts with treated ribbons fitted around eave spaces is unlikely, further reducing the risk of touch or ingestion by children, hence providing greater safety profiles (Fig. 1). Additional studies will be necessary to ascertain the safety and efficacy of each chemical treatment and doses used on the ribbons.

To maximize impact, it is best to treat the ribbons using insecticides or combinations of insecticides with multiple modes of action (toxicity, spatial repellency and feeding inhibition). This may include vapour-phase insecticides such as transfluthrin which is currently used in most applications, or contact insecticides to ensure that mosquitoes attempting to enter houses via eaves can be directly killed or incapacitated. Using insecticides with toxicant and repellent effects also reduces the likelihood that mosquitoes are diverted to non-users [43, 45]. This way, even non user households can accrue significant communal protection, resulting from the mass killing effects of the product in the user households [40]. Furthermore, the eave ribbons present a potential environmentally-friendly vector control tool due to its biodegradability features; and it is unlikely that they would require more extensive disposal methods than the standard vector control methods, i.e. ITNs and IRS. Nonetheless, additional studies are necessary to assess whether alternative substrates, could also be used in place of hessian for manufacturing the reave ribbons so as to reduce delivery costs and maximize efficacy and longevity.

### Comparative evaluation of the insecticide-treated eave ribbons and indoor residual spraying

While IRS remains one of the most efficacious vector control tools, its deployment and overall impact are increasingly limited to small geographic areas due to multiple factors, notably high costs and logistical challenges (Table 2). Going forward, it is important to investigate improved approaches to sustain the efficacy and overall impact IRS while maximizing scale, coverage and affordability. One option already tested involved partial spraying of IRS on a section of walls [58], which effectively reduces the pesticide quantities but not the other difficulties such as the need to remove people’s belongings before spraying.

Given the simplicity and likely scalability of eave ribbons for delivering effective insecticides targeted at the eave space, it is reasonable to also comparatively assess the performance of the eave ribbons and IRS. The outcomes of such comparative evaluations would enable determination of whether the ribbons could constitute a less-cumbersome but more-affordable substitute for IRS by using lower quantities of insecticides near eaves to maximise efficacy.

Similar to standard IRS, the eave ribbons can be treated with different insecticides, singly or in combination, which would enable careful selection and deployment for to manage resistance [50]. They may also constitute a portable insecticidal surfaces with same functionality of mass-killing mosquitoes destined indoors, with the added advantage of being distributable as pre-treated fabrics for easy fitting onto user homes. This would increase scalability and provide additional advantages over standard IRS, which though highly impactful, is still deployed to far fewer households than ITNs and usually with limited adherence to the WHO resistance management guidelines [59].

Given the ease-of-use and affordability (current unsubsidized prototype estimates are ~ $7.00/house/year), eave ribbons may cost-effectively protect entire households both indoors and outdoors [37, 39]), possibly expanding the protective coverage beyond level currently achievable with standard IRS. Lessons from ITN distribution campaigns can be adapted to support such operations including supply chain and transform eave ribbons into a viable alternatives to IRS. Further development of the eave ribbons should address context-specific challenges to optimize efficacy, reduce costs for manufacturing, delivery and installation and further enhance both simplicity and scalability. It will also be important to explore the supply chain determinants relevant to this product and what it would take to achieve the perceived scalability.

To validate this potential, studies should be conducted to directly compare protective efficacy and effectiveness of insecticide-treated eave ribbons relative to standard IRS. While full-scale epidemiological studies (e.g. randomised controlled trials) would be desirable, experimental hut studies complemented with small-scale village trials measuring entomological outcomes could already provide reasonable indications of the potential of the ribbons to impact vector densities and transmission intensities (Table 2).

### Effective stakeholder engagement will be essential for further development and scale-up of eave ribbons

The need for stakeholder engagement is increasingly being recognized as an essential component in malaria control efforts [62, 63]. It is essential that proponents of any new interventions consider views and opinions of key stakeholders early on in the development of these interventions to ensure that they are affordable, acceptable, and are responsive to the needs of the targeted users. In the case of eave ribbons, baseline assessment of the need and potential of this intervention appears promising but there still needs to be additional engagement. A study by Swai et al. [39] indicated high levels of acceptance for this technology among community members in south-eastern Tanzania; approximately 90% of community members reported willingness to use the ribbons and were willing to pay up to $4.3 for the ribbons. In a separate study by Finda et al. [64], which compared perceptions of a range of stakeholder groups regarding the potential of several malaria control interventions for malaria control and elimination strategies, spatial repellents such as those delivered by eave ribbons were among the most preferred. Some advantages of this technology included the perceived ease-of-use, affordability and ease-of-access. This baseline knowledge on responses from potential users is important in developing products fitting the needs and preferences of target communities.

Eave ribbons offer a practical and affordable intervention suitable for all households, without requiring any major house modifications, electricity and sophisticated skill. There are opportunities to engage local groups at various stages of development, treatment, deployment or maintenance of the ribbons. Involvement of groups such as local tailors, women groups and local entrepreneurs will not only improve ownership, but will also provide direct employment in the communities. Such practices are already being implemented at small scale by Ifakara Health Institute, and could be expanded to support scaled-up distribution campaign.

For greater effectiveness, inter-sectoral collaboration is also important in the scale up of the eave ribbons technology. Partnerships between the ministry of health, ministry of housing, chemical providers and local leaders is necessary for scale-up and sustainability of these eave ribbons across the country. It would be important to adapt some approaches such as those proven effective for advocacy, social mobilization and legislative change to improve outcomes in vector control [65].

### Potential pathways for development, evaluation, pre-qualification and adoption of eave ribbons

The WHO has outlined the steps required in evaluation of new vector control interventions [66]. If an intervention falls into a class already covered by existing WHO guidelines, the particular intervention should be assigned to pre-qualification pathway so as to assess its safety, quality and entomological efficacy without requiring additional epidemiological studies [66]. On the other hand, interventions not fitting an established class should be backed by at least two large scale trials with clinical outcomes.

One plausible pathway would be to present the eave ribbons as possible substitute for IRS, and assume the existing public health value, as supported by the current evidence [67, 68]. This would be particularly applicable for ribbons treated with contact insecticides, and would depend on data from the comparative studies proposed above. Depending on final versions, another alternative pathway would be to consider this a new intervention class and seek epidemiological evidence e.g. from cluster randomised controlled trials. Other options may be to consider this in the same class as lethal house lures [69] or spatial repellents [70], for which epidemiological evidence is either partially available or is underway. Unlike eave tubes, the ribbons can be fitted onto any house type and do not require any additional construction to fill up the eave spaces. However, since both of them primarily target mosquitoes entering houses through eaves developers and regulatory agencies may consider including the eave ribbons in the same class. On the other hand, the current class of spatial repellents does not restrict the choice of delivery formats. Therefore, eave ribbons treated using vapour-phase insecticides conferring spatial-repellent effects (e.g. transfluthrin) could potentially be classified as such. Lastly, developers may consider this product as a niche intervention to be deployed in specific local contexts following local regulatory approvals, but without necessarily going through the WHO pathways.

Whichever path is taken, additional evaluations with either entomological or clinical outcomes will be necessary to inform adoption in different contexts, and to explore the supply chain factors relevant to the product and its perceived scalability.

## Conclusions

This article reviewed the evidence for efficacy of eave ribbons and discussed their potential as a supplementary malaria control tool with added advantages of being affordable, locally sustainable, easy-to-target and versatile and effective. The review excludes several other eave-based technologies, and instead focuses primarily on the hessian-based eave ribbons technology conferring protection both indoors and outdoors. The eave ribbons can be treated with vapour-phase pyrethroids such as transfluthrin (which can kill *Anopheles* mosquitoes, repel them over wide areas and inhibit blood-feeding) or contact non-pyrethroid insecticides such clothianidin, bendiocarb or pirimiphos methyl, currently approved for IRS. The technology exploits the natural house-entry behaviours of malaria vectors to deliver mosquitocidal or repellent actives around eave spaces, and can prevent mosquito bites indoors and in outdoor spaces. While malaria programmes must seek long-term approaches to sustain the gains accrued from current tools, technologies such as eave ribbons could enable judicious application of available insecticides in ways that maximize transmission control even in the lowest-income communities. Eave ribbons could, therefore, have potential as a supplementary tools to address gaps associated with ITNs and IRS. Given their simplicity and demonstrated potential in previous studies, future research should investigate ways to optimize scalability and effectiveness of the ribbons. It is also important to assess whether the ribbons may constitute a less-cumbersome but more-affordable substitute for other interventions such as IRS; by judiciously using lower quantities of selected insecticides targeted around eave spaces to deliver equivalent or greater suppression of malaria transmission.

## Data Availability

Not applicable.

## Abbreviations

ATSBs: Attractive targeted sugar baits
EU: European Union
GTS: Global Technical Strategy
IRS: Indoor residual spraying
ITNs: insecticide-treated nets
SR: Spatial repellents
OBTs: Odour-baited traps
WHO: World Health Organization
